# Prevalence and risk factors of cholelithiasis in patients with spinal cord injury: A cross-sectional analysis

**DOI:** 10.1371/journal.pone.0344816

**Published:** 2026-03-13

**Authors:** Rongfu Fan, Xinqi Cao, Zhihua Long, Bobo Ma, Qing Xu, Dejian Zhang

**Affiliations:** 1 Department of General Surgery, Beijing Bo’ai Hospital, China Rehabilitation Research Center, Beijing, China; 2 School of Rehabilitation Medicine, Capital Medical University, Beijing, China; 3 Department of General Surgery, Beijing Di’tan Hospital, Capital Medical University, Beijing, China; 4 Department of Emergency, Beijing Bo’ai Hospital, China Rehabilitation Research Center, Beijing, China; University of Diyala College of Medicine, IRAQ

## Abstract

**Objective:**

The purpose of the present study was to investigate the incidence of cholelithiasis and evaluate the risk factors for cholelithiasis in patients with spinal cord injury (SCI).

**Methods:**

We analyzed 1,530 SCI patients (1,186 males and 344 females) from the China Rehabilitation Research Center (2010–2019) and compared patients with cholelithiasis (n = 289) and without cholelithiasis. The variables included age, gender, marital status, blood glucose level, motor function, and American Spinal Injury Association (ASIA) Impairment Scale (AIS) score.

**Results:**

A total of 1530 patients with SCI, including 1186 males and 344 females, were included in this study. The prevalence of cholelithiasis was 18.89%. Univariate analysis revealed significant differences in age (P < 0.001), marital status (P < 0.001), blood glucose level (P < 0.001), and preserved motor function (P = 0.033). Multivariate analysis revealed the following independent risk factors: age ≥ 50 years (OR=1.637; 95% CI = 1.016–2.639; P = 0.043), being married (OR=1.902; 95% CI = 1.061–3.411; P = 0.031), lack of motor function (OR=1.587; 95% CI = 1.194–2.111; P < 0.001), and hyperglycemia (OR=1.764; 95% CI = 1.164–2.673; P = 0.007). Gender, lipid levels, SCI segment, and AIS grade were not significantly different.

**Conclusion:**

Patients with SCI have a higher incidence of gallstones. Multivariate logistic regression analysis revealed that the occurrence of cholelithiasis in patients with SCI is closely related to age, marital status, blood glucose level and motor function preservation. Tailored preventive and therapeutic approaches should be developed for SCI patients, with intensified monitoring and intervention for high-risk patients to significantly improve quality of life.

## Introduction

Spinal cord injury (SCI) is a serious central nervous system disease triggered by various causes, with manifestations of motor, sensory, sphincter and autonomic nerve dysfunction below the level of injury, and it is still a medical problem worldwide. Some digestive tract problems subsequently become increasingly obvious, including constipation, abdominal distension, changes in gastrointestinal motility and an increased incidence of gallstones [1–4]. According to some studies in North America [5–9], the incidence of cholelithiasis in patients with SCI is significantly greater than that in the normal population; thus, SCI is considered a risk factor for cholelithiasis.

Therefore, effective prevention and management of cholelithiasis in patients with SCI represents a critical research priority. With the advancement of technology and society, patients with spinal cord injuries have a life expectancy similar to that of healthy individuals. Accidental injuries can be a turning point for both the individual and their family. Previous studies in China have focused primarily on the male population, while research on cholelithiasis, in patients with SCI, particularly large-scale studies, remains limited. Existing domestic studies are limited by small sample sizes and heterogeneous patient populations, making their findings difficult to generalize. To address this gap, we conducted a retrospective analysis of electronic medical records from 1,530 SCI patients treated at the China Rehabilitation Research Center over a 10-year period, aiming to investigate both the incidence and risk factors for cholelithiasis in this population. Patient surveys based on the China Rehabilitation Research Center provide new data support to study the characteristics of gallstones in patients with SCI in China, thereby providing a reference for the prevention and treatment of cholelithiasis in this unique group.

## Methods

### Research subjects

SCI inpatients at the China Rehabilitation Research Center from 1/1/2010–31/12/2019 were selected. The inclusion criterion was as follows: (1) age ≥ 18 years; and (2) complete medical records, hepatobiliary ultrasound and/or abdominal CT results. The exclusion criteria were as follows: (1) age < 18 years; (2) conus cauda equina injury; (3) foreign nationality patients; (4) patients who simultaneously suffer from major diseases, such as tumors and arteriovenous malformations; (5) patients who experience cholelithiasis before SCI; (6) patients who have undergone cholecystectomy; (7) patients who refuse to undergo relevant clinical and imaging examinations; and (8) incomplete relevant clinical data. In total, 1530 patients met the inclusion criteria.

### Data collection

The present study was reviewed and approved by the Ethics Committee of Beijing Boai Hospital (Ethics Approval No. EC-RF-SC-010–02); the requirement for written informed consent was waived by the Ethics Committee.

Although the authors had access to potentially identifiable personal information during data collection and processing, all data were fully anonymized prior to analysis. Data access for research purposes was obtained on December 8, 2020.

Patients who were in a fasting state and positioned supine (with lateral decubitus positioning if necessary) underwent multiangle examinations of the liver, gallbladder, spleen, and pancreas using color Doppler ultrasound or computed tomography (CT). On the basis of the imaging findings, participants were categorized into either the cholelithiasis group or the noncholelithiasis (control) group. Patients in the cholelithiasis group were diagnosed with cholelithiasis on the basis of the following criteria: ultrasound indicated high echogenicity in the gallbladder area accompanied by a shadow on ultrasound that moved with body position [10]; the bile duct inside and outside the liver showed a strong light mass shadow, accompanied by a shadow on ultrasound; and CT indicated low- or high-density calculi in the gallbladder and bile duct. The noncholelithiasis group included patients who did not meet the above criteria for cholelithiasis. Clinicians collected general demographic data, obtained blood samples for biochemical analysis, and electronically recorded and organized all the information.

### Study variables

On the basis of the study objectives and a review of the literature, the following variables for data collection were determined through discussion: (1) demographic characteristics potentially associated with cholelithiasis risk, including age, gender, and marital status; (2) biochemical parameters, including fasting plasma glucose (FPG); lipid profiles, including triglyceride (TG), total cholesterol (TC), high-density lipoprotein cholesterol (HDL-C), and low-density lipoprotein cholesterol (LDL-C) levels; and (3) clinical data, including the degree of SCI, ASIA Impairment Scale (AIS) grade, and abdominal ultrasound/CT results.

Grouping of variables was performed on the basis of cutoff values. Blood glucose grouping was performed according to diabetes diagnostic criteria [10]; FPG ≥ 7.0 mmol/L was defined as hyperglycemia, while FPG < 7.0 mmol/L was defined as nonhyperglycemic. The lipid cutoff values were as follows: TC concentration of 5.7 mmol/L; TG concentration of 1.73 mmol/L; LDL-C concentration of 3.1 mmol/L; and HDL-C concentration of 0.9 mmol/L [11]. The AIS grading was as follows: AIS A (complete impairment), characterized by no sensory or motor function preserved in sacral segments S4–S5; AIS B (incomplete sensory impairment), characterized by sensory function, but not motor function, preserved below the neurological level of injury (NLI) and includes sacral segments S4–S5; AIS C (incomplete motor impairment; majority < grade 3), characterized by motor function preserved below the NLI, with a muscle grade less than 3 in more than half of the key muscles below the NLI; AIS D (incomplete motor impairment; at least half ≥ grade 3), characterized by motor function preserved below the NLI, with a muscle grade of 3 or greater in at least half of the key muscles below the NLI; and AIS E (normal), characterized by normal sensory and motor functions. The variable assignments are provided in S1 Table.

### Statistical analysis

Statistical analyses were performed using SPSS version 24.0. Categorical variables are presented as frequencies and percentages, with between-group comparisons analyzed using the chi-square test. Continuous variables are expressed as the mean ± standard deviation (mean ± SD), with between-group comparisons assessed by independent samples t tests. Binary logistic regression was employed to identify risk factors associated with cholelithiasis. A two-sided P value < 0.05 was considered to indicate statistical significance.

## Results

### Baseline characteristics

A total of 1,530 subjects were enrolled, comprising 1,186 males (77.52%) and 344 females (22.48%). Among these, 289 individuals were diagnosed with cholelithiasis, yielding a prevalence rate of 18.89% (S2 Table).

### Univariate logistic analysis

Chi-square analysis revealed statistically significant associations between the occurrence of cholelithiasis in patients with SCI and several other factors (p < 0.05). Significant differences were observed in age distribution (χ2 = 26.802, p < 0.001); the highest prevalence of cholelithiasis was in the ≥ 50-year-old group (42.21%), whereas the lowest prevalence of cholelithiasis was in the 30–39-year-old group (13.49%). Regarding marital status, the proportion of married individuals was significantly higher in the cholelithiasis group than in the non-cholelithiasis group (93.08% vs. 84.37%; χ2 = 14.074; p < 0.001). In terms of motor function, patients with complete motor injuries (AIS A/B) accounted for a significantly larger proportion of the cholelithiasis group compared to the non-cholelithiasis group (67.82% vs. 60.84%; χ2 = 4.566; p = 0.033). Additionally, the proportion of individuals with FPG ≥ 7.0 mmol/L was significantly higher in the cholelithiasis group than in the non-cholelithiasis group (13.15% vs. 6.93%; χ2 = 11.352; p < 0.001).

No significant differences were observed for sex (p = 0.391), NLI (p = 0.809), AIS grade (p = 0.07), or lipid profile indicators (TG: p = 0.405; TC: p = 0.816; HDL-C: p = 0.542; LDL-C: p = 0.402). Although the prevalence of fatty liver disease was slightly lower in the cholelithiasis group than in the noncholelithiasis group (20.07% vs. 24.98%), this difference did not reach statistical significance (p = 0.092). These results suggested that age, marital status, motor function, and hyperglycemia (FPG ≥ 7.0 mmol/L) are significantly associated with gallstone occurrence (S2 Table).

### Multivariate analysis

**Fig 1:**
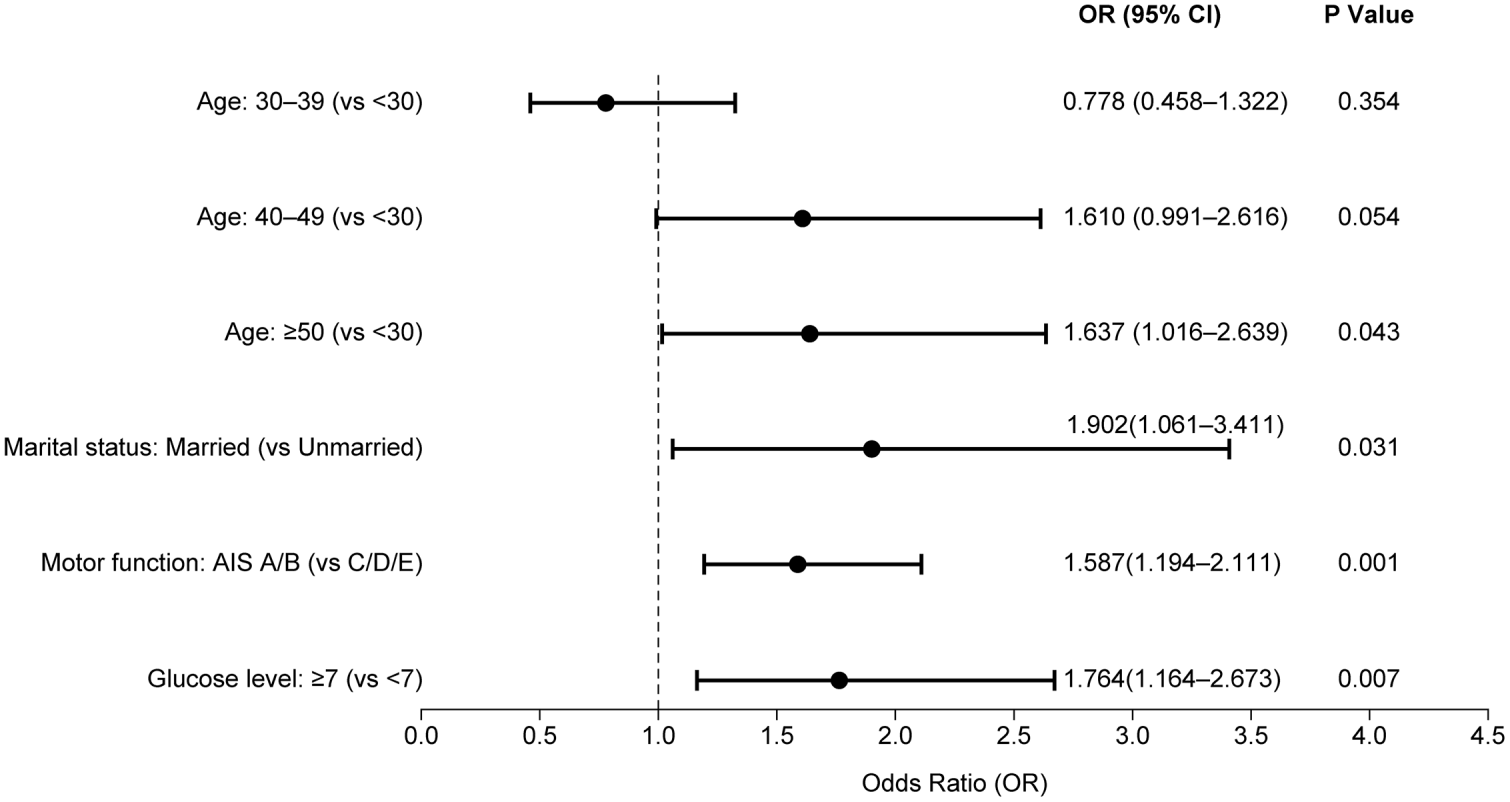
Forest plot of multivariate logistic regression analysis.

Variables demonstrating statistical significance in the univariate analysis were evaluated by multivariate logistic regression analysis. The forest plot shown in Fig 1 illustrates several independent predictors associated with the occurrence of cholelithiasis in patients with SCI. Age ≥ 50 years, marital status, complete motor injury status, and a FPG concentration ≥7.0 mmol/L were independent risk factors for gallstone development in patients with SCI (all P < 0.05). Compared with individuals aged <30 years, those aged ≥50 years had a 63.7% higher risk (OR = 1.637; 95% CI: 1.016, 2.639). Compared with unmarried individuals, married individuals had a 90.2% greater risk (OR = 1.902; 95% CI: 1.061, 3.411). Complete motor injury was associated with a 58.7% increase in risk (OR = 1.587; 95% CI: 1.194, 2.111). Compared with those with FPG < 7.0 mmol/L, participants with FPG ≥ 7.0 mmol/L had a 76.4% higher risk (OR = 1.764; 95% CI: 1.164; 2.673).

Forest plot of multivariable logistic regression analysis of risk factors associated with cholelithiasis in patients with SCI. The plot displays the odds ratios (ORs) and 95% confidence intervals (CIs) for each variable. Factors on the right side of the line (OR > 1) represent increased risk, whereas those on the left (OR < 1) indicate protective effects.

**Fig 2:**
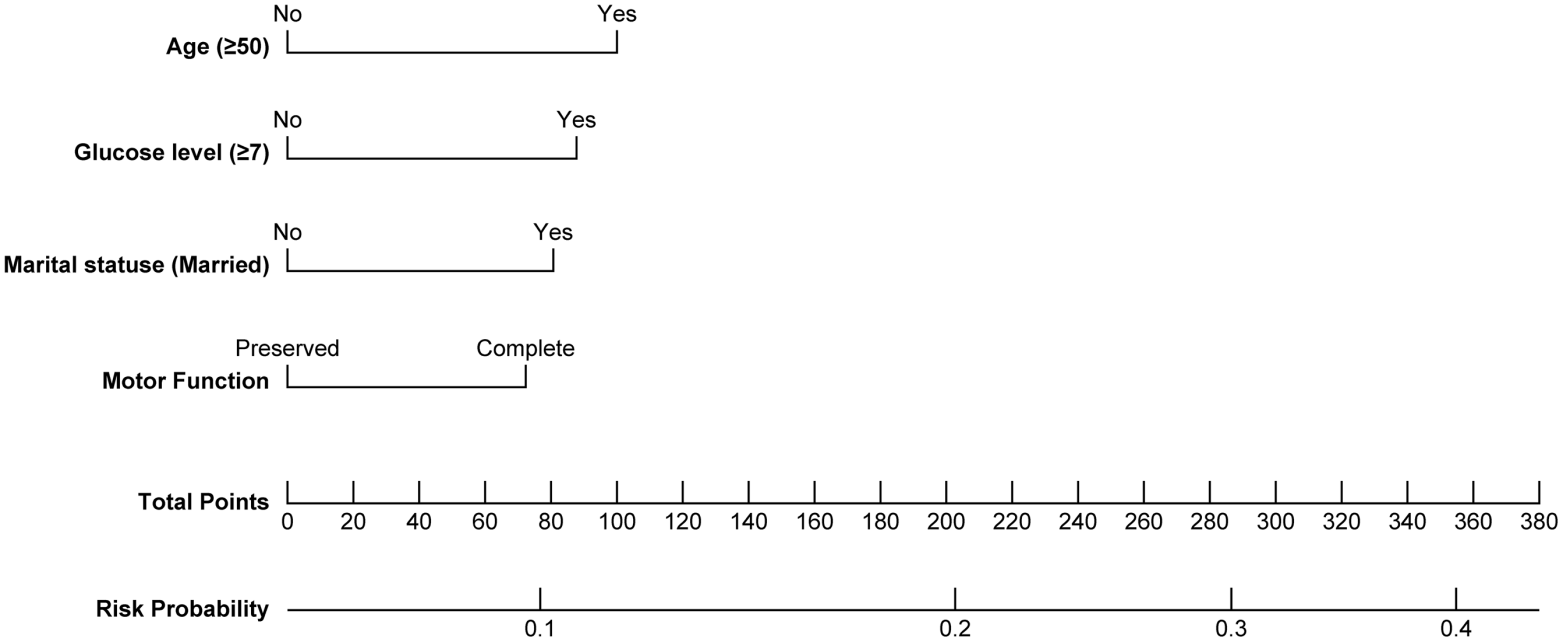
Nomogram for predicting cholelithiasis in patients with SCI.

**Fig 3:**
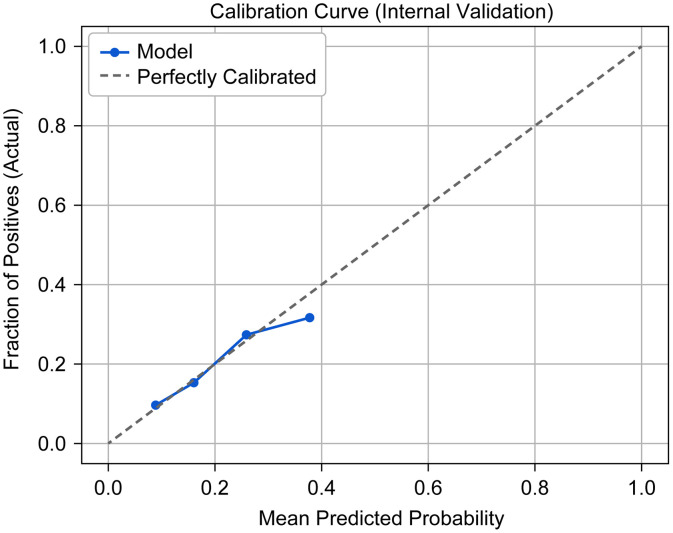
Calibration curve (internal validation).

On the basis of the independent risk factors identified in the multivariable logistic regression analysis, including age, motor function status, and FBG (≥ 7 mmol/L), a clinical prediction nomogram was developed to estimate the individual risk of cholelithiasis in patients with SCI (Fig 2). The nomogram allows for a quantitative assessment by summing the scores of each predictor. The model demonstrated stable performance during internal validation using the bootstrap method (1,000 repetitions). The original area under the receiver operating characteristic curve (AUC) was 0.628, with a bootstrap-corrected AUC of 0.625 (95% CI: 0.614–0.632), indicating modest but reliable discriminative ability. Furthermore, the Brier score was 0.148, reflecting a high level of overall predictive accuracy. The calibration plot (Fig 3) revealed excellent agreement between the predicted probabilities and the actual observed outcomes, confirming the reliability of the model for clinical risk stratification.

## Discussion

The present study included 1530 patients with SCI, including 1186 males and 344 females, and the prevalence of cholelithiasis was 18.89%. Univariate analysis revealed significant differences in age, marital status, blood glucose level, and motor function preservation. Multivariate analysis revealed that a higher age, being married, a lack of motor function, and hyperglycemia were independent risk factors for cholelithiasis. Gender, lipid levels, SCI segment, and AIS grade were not significantly different.

Cholelithiasis is a common condition in general surgery, with an increasing incidence associated with improved living standards. Patients with SCI represent a distinct population. After SCI, a series of digestive tract problems, such as defecation disorders, abdominal distension, and gallstone disease, occur [12]. North American studies have indicated a higher prevalence of cholelithiasis in SCI patients (up to 19.6%) than in the general population [3]. Xia et al. [6] reported a 26.0% prevalence among 100 male SCI patients. In the present study, the prevalence of cholelithiasis was 18.9% among 1,530 SCI patients. The increased risk of cholelithiasis may result from post-SCI pathophysiological changes, such as abnormal gastrointestinal motility, impaired gallbladder function, disrupted enterohepatic circulation, altered gallbladder emptying, and modified bile composition [15–18]. Thomas et al. [19] reported that slowing colonic transmission is related to increased plasma deoxycholic acid levels. Patients with SCI have neurogenic intestinal dysfunction, slowed intestinal transport, and constipation, which may be among the mechanisms underlying the high incidence of cholelithiasis in patients with SCI. In addition, the neuroregulation and humoral regulation of intestinal movement dysfunction, intestinal motility disorders, and intestinal flora disorders are prone to occur after SCI [20–22]. Such changes can disrupt the enterohepatic circulation of bile acids, resulting in cholesterol supersaturation that increases the risk of cholelithiasis [23–25].

The present results suggested that age is a risk factor for cholelithiasis in patients with SCI. Age is also a risk factor for gallstone disease in the general population [1,4]. Most studies have revealed that the incidence of cholelithiasis varies across different ages and that the incidence of cholelithiasis increases in individuals over 40 years old [7]. This phenomenon may be attributed to age-related decreases in hepatic LDL receptor activity, which increase biliary cholesterol saturation [8–10]. Furthermore, aging reduces gallbladder contractility and delays bile emptying. In patients with SCI, autonomic dysfunction exacerbates this phenomenon, promoting bile stasis [13]. The progressive limitation of mobility in elderly SCI patients additionally diminishes the facilitatory effect of positional changes on gallbladder emptying, further contributing to age as a significant risk factor for cholelithiasis development in this population [14].

The present study revealed that the incidence of cholelithiasis was significantly greater among married patients than among unmarried patients (P = 0.031). Combined with the findings of the present study and the evidence in the literature, there are several possible mechanisms. First, age is a confounding effect. The married patients were older in the present study (47.3 ± 1.39 years old vs. 42.6 ± 1.43 years in the unmarried group), and increasing age is a known risk factor for cholelithiasis [7]. Therefore, marital status may indirectly influence cholelithiasis development through the age variable. Second, lifestyle and behavioral differences may contribute to this difference in cholelithiasis risk. Married individuals may reduce physical activity because of increased family responsibility. Studies have shown that a lack of exercise is closely associated with decreased gallbladder systolic function, which subsequently triggers cholestasis. In addition, married individuals are more likely to have a high-calorie, high-cholesterol diet, with the average BMI of married men being 1.3 kg/m² greater than that of unmarried men. An increased BMI is significantly associated with the occurrence of cholelithiasis. Third, psychological stress and metabolic disorders may contribute to the increased risk of cholelithiasis. Married individuals may face greater family and financial stress. Long-term psychological stress activates the hypothalamic‒pituitary‒adrenal axis (HPA axis), leading to increased cortisol levels, which in turn affects lipid metabolism and bile components [26]. In conclusion, marital status may influence cholelithiasis risk through multiple pathways, including age, lifestyle, and psychological stress. This association still needs to be verified by more longitudinal studies to clarify the causality and exclude the influence of confounding factors.

The present study revealed that a lack of motor function preservation was a significant risk factor for cholelithiasis in patients with SCI. The underlying mechanisms likely involve autonomic dysfunction and restricted mobility effects. With regard to autonomic dysfunction, complete injuries above the T6 level disrupt the sympathetic–vagal balance, resulting in impaired gallbladder contractility [13]. Regarding restricted mobility effects, the complete loss of motor function eliminates the facilitatory effect of positional changes on gallbladder emptying, thereby establishing motor completeness as a critical determinant of cholelithiasis risk in this population.

With respect to the history of treatment for cholelithiasis in patients with SCI, many strategies have been explored, such as prophylactic surgery. The current point of view, treatment measures and individuals remain the same.

Because the present study was a single-center retrospective study, a multicenter study with a relatively large sample size should be conducted in the future to validate the findings and improve the general applicability of the results. More potential variables (intestinal flora, bile acid metabolism indicators, etc.) should be included in future studies to further clarify the relationships between cholelithiasis and various risk factors. Predictive analysis revealed a significant protective effect against cholelithiasis in patients with SCI aged 30–39 years who were unmarried, whose motor function was preserved, and who maintained normal fasting plasma glucose levels (<7.0 mmol/L). In response to the need for a practical tool in clinical settings, we integrated key predictors into a nomogram to provides a personalized risk profile for patients with SCI, facilitating early screening for cholelithiasis. The present model highlights the significant roles of age and metabolic factors (such as FBG) alongside physical impairment (motor function). Although the AUC of 0.625 suggests modest discrimination, it is consistent with the multifactorial and complex nature of cholelithiasis in SCI patients. Moreover, the Brier score (0.148) and the well-aligned calibration curve indicated that the model is statistically calibrated, indicating that the predicted risks are close to the actual event rates.

## Conclusion

In summary, patients with SCI have a higher incidence of cholelithiasis than healthy individuals, and the risk factors for cholelithiasis in patients with SCI include age, marital status, complete motor injury, and high blood glucose levels. These findings can be applied to develop evidence-based cholelithiasis prevention and control strategies for patients with SCI while laying a foundation for the personalized identification of relevant high-risk individuals.

**Key points**

**Question**

The present study investigated the prevalence of cholelithiasis in patients with spinal cord injury (SCI) and evaluated the associated risk factors.

**Findings**

Among 1,530 SCI patients, the cholelithiasis prevalence was 18.89% Multivariate analysis revealed that advanced age, marital status, no preserved motor function, and elevated blood glucose levels were associated with cholelithiasis in SCI patients.

**Meaning**

The present study revealed that the incidence of cholelithiasis was significantly greater in married patients than in unmarried patients (P = 0.031).

## Supporting information

S1 Table Variable assignment. (DOCX)

S2 Table Comparison of baseline characteristics and univariate analysis of risk S1 factors. (DOCX)

S1 Fig Forest plot of multivariate logistic regression analysis. (TIF)

S2 Fig Nomogram for predicting cholelithiasis in patients with SCI. (TIF)

S3 Fig Calibration curve (internal validation). (TIF)
