# Supplementary material for: Prevalence and risk factors of cholelithiasis in patients with spinal cord injury: A cross-sectional analysis
Source: PLoS One. 2026 Mar 13;21(3):e0344816. doi: 10.1371/journal.pone.0344816 (PMC12987457; doi:10.1371/journal.pone.0344816)
Supplement: S1 Table — (DOCX) [file pone.0344816.s001.docx]

| Variable | Assignment |
| --- | --- |
| Sex  Age  Marital status  AIS grade  NLI  Motor function  Cholelithiasis  Fatty liver  Fasting blood glucose  TC (mmol/L)  TG (mmol/L)  LDL (mmol/L)  HDL (mmol/L) | Male = 1; Female =2  "<30"=1; "30-39"=2; "40-49"=3; "≥50"=4  Unmarried = 1; Married =2  A=1; B=2; C=3; D=4; E=5  "<T10"=1;"≥T10"=2  AIS A/B= 1; AIS C/D/E =0  Yes = 1; No =2  Yes = 1; No =2  "≥ 7" = 1; "<7" =2  "≥5.7"=1; "<5.7"=2  "≥1.73"=1; "<1.73"=2  "≥3.1"=1; "<3.1"=2  "≥0.9"=1; "<0.9"=2 |
