# Supplementary material for: Prevalence and risk factors of cholelithiasis in patients with spinal cord injury: A cross-sectional analysis
Source: PLoS One. 2026 Mar 13;21(3):e0344816. doi: 10.1371/journal.pone.0344816 (PMC12987457; doi:10.1371/journal.pone.0344816)
Supplement: S2 Table — (DOCX) [file pone.0344816.s002.docx]

| Variable | Classification | Non-cholelithiasis group (n=1241) | Cholelithiasis group (n=289) | Total | χ^2^ | p |
| --- | --- | --- | --- | --- | --- | --- |
| Sex | Male | 956 (77.03%) | 230 (79.58%) | 1186 (77.52%) | 0.734 | 0.391 |
|  | Female | 285 (22.97%) | 59 (20.42%) | 344 (22.48%) |  |  |
| Age | <30 | 282 (22.72%) | 40 (13.84%) | 322 (21.05%) | 26.802 | <0.001 |
|  | 30~39 | 263 (21.19%) | 39 (13.49%) | 302 (19.74%) |  |  |
|  | 40~49 | 284 (22.88%) | 88 (30.45%) | 372 (24.31%) |  |  |
|  | ≥50 | 412 (33.20%) | 122 (42.21%) | 534 (34.90%) |  |  |
| Marital status | Unmarried | 194 (15.63%) | 20 (6.92%) | 214 (13.99%) | 14.074 | <0.001 |
|  | Married | 1047 (84.37%) | 269 (93.08%) | 1316 (86.01%) |  |  |
| NLI | <T10 | 1032 (83.16%) | 238 (82.35%) | 1270 (83.01%) | 0.058 | 0.809 |
|  | ≥T10 | 209 (16.84%) | 51 (17.65%) | 260 (16.99%) |  |  |
| AIS  grade | A | 574 (46.25%) | 150 (51.90%) | 724 (47.32%) | 7.063 | 0.07 |
|  | B | 180 (14.50%) | 46 (15.92%) | 226 (14.77%) |  |  |
|  | C | 199 (16.04%) | 46 (15.92%) | 245 (16.01%) |  |  |
|  | D | 288 (23.21%) | 47 (16.26%) | 335 (21.90%) |  |  |
| Motor function | AIS A/B | 755 (60.84%) | 196 (67.82%) | 951 (62.16%) | 4.566 | 0.033 |
|  | AIS C/D/E | 486 (39.16%) | 93 (32.18%) | 579 (37.84%) |  |  |
| Fatty liver | yes | 310 (24.98%) | 58 (20.07%) | 368 (24.05%) | 2.831 | 0.092 |
|  | no | 931 (75.02%) | 231 (79.93%) | 1162 (75.95%) |  |  |
| Blood glucose | <7 | 1155 (93.07%) | 251 (86.85%) | 1406 (91.90%) | 11.352 | <0.001 |
|  | ≥7 | 86 (6.93%) | 38 (13.15%) | 124 (8.10%) |  |  |
| TG (mmol/L) | <1.73 | 871 (70.19%) | 195 (67.47%) | 1066 (69.67%) | 0.692 | 0.405 |
|  | ≥1.73 | 370 (29.81%) | 94 (32.53%) | 464 (30.33%) |  |  |
| TC (mmol/L) | <5.7 | 1136 (91.54%) | 265 (91.70%) | 1401 (91.57%) | 0.054 | 0.816 |
|  | ≥5.7 | 105 (8.46%) | 24 (8.30%) | 129 (8.43%) |  |  |
| HDL-C (mmol/L) | <0.9 | 591 (47.62%) | 144 (49.83%) | 735 (48.04%) | 0.372 | 0.542 |
|  | ≥0.9 | 650 (52.38%) | 145 (50.17%) | 795 (51.96%) |  |  |
| LDL-C (mmol/L) | <3.1 | 846 (68.17%) | 189 (65.40%) | 1035 (67.65%) | 0.702 | 0.402 |
|  | ≥3.1 | 395 (31.83%) | 100 (34.60%) | 495 (32.35%) |  |  |
