# Supplementary figures and images for: Prevalence and risk factors of cholelithiasis in patients with spinal cord injury: A cross-sectional analysis
Source: PLoS One. 2026 Mar 13;21(3):e0344816. doi: 10.1371/journal.pone.0344816 (PMC12987457; doi:10.1371/journal.pone.0344816)

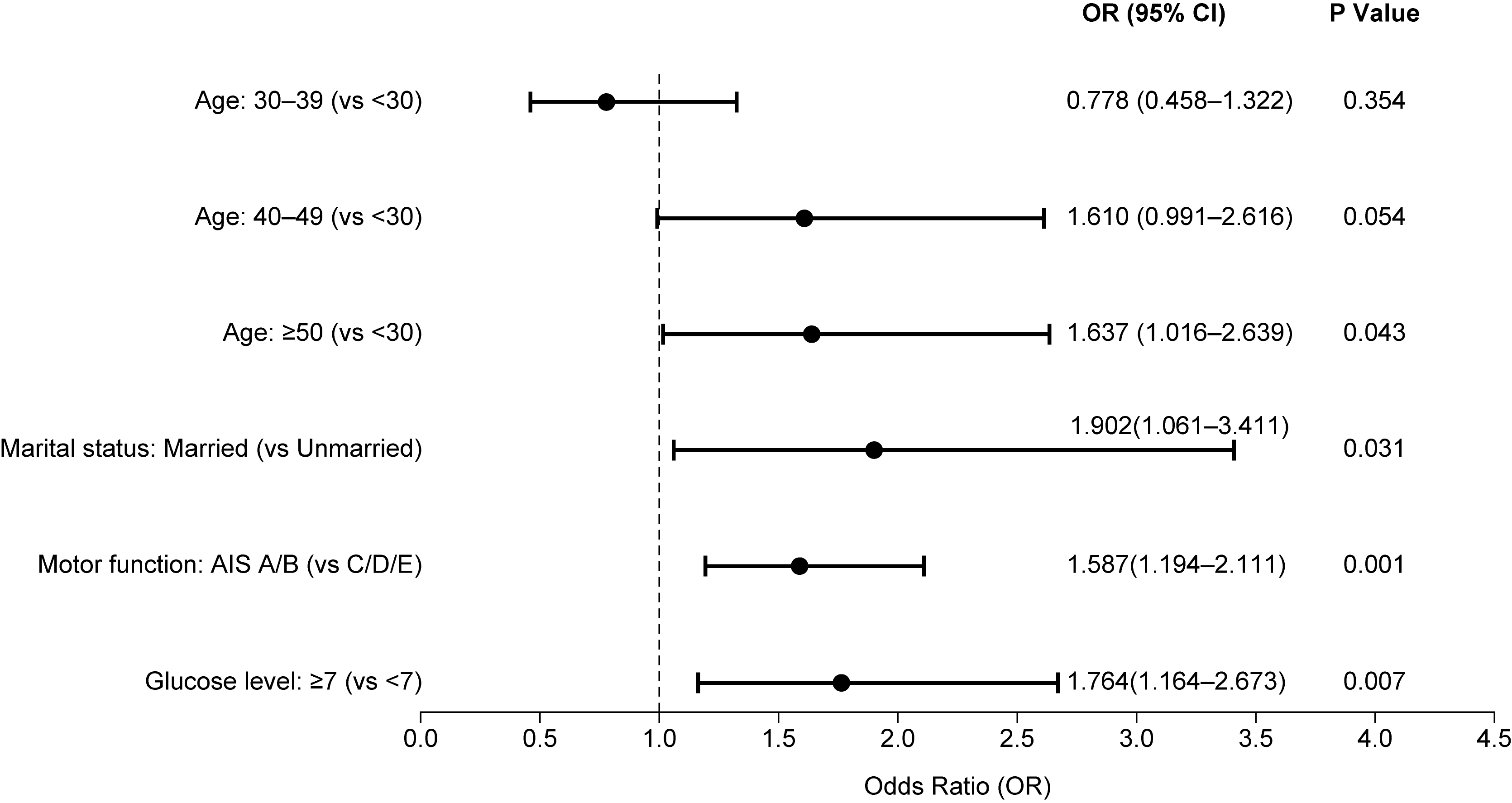

Supplement: S1 Fig — (TIF) [file pone.0344816.s003.tif]

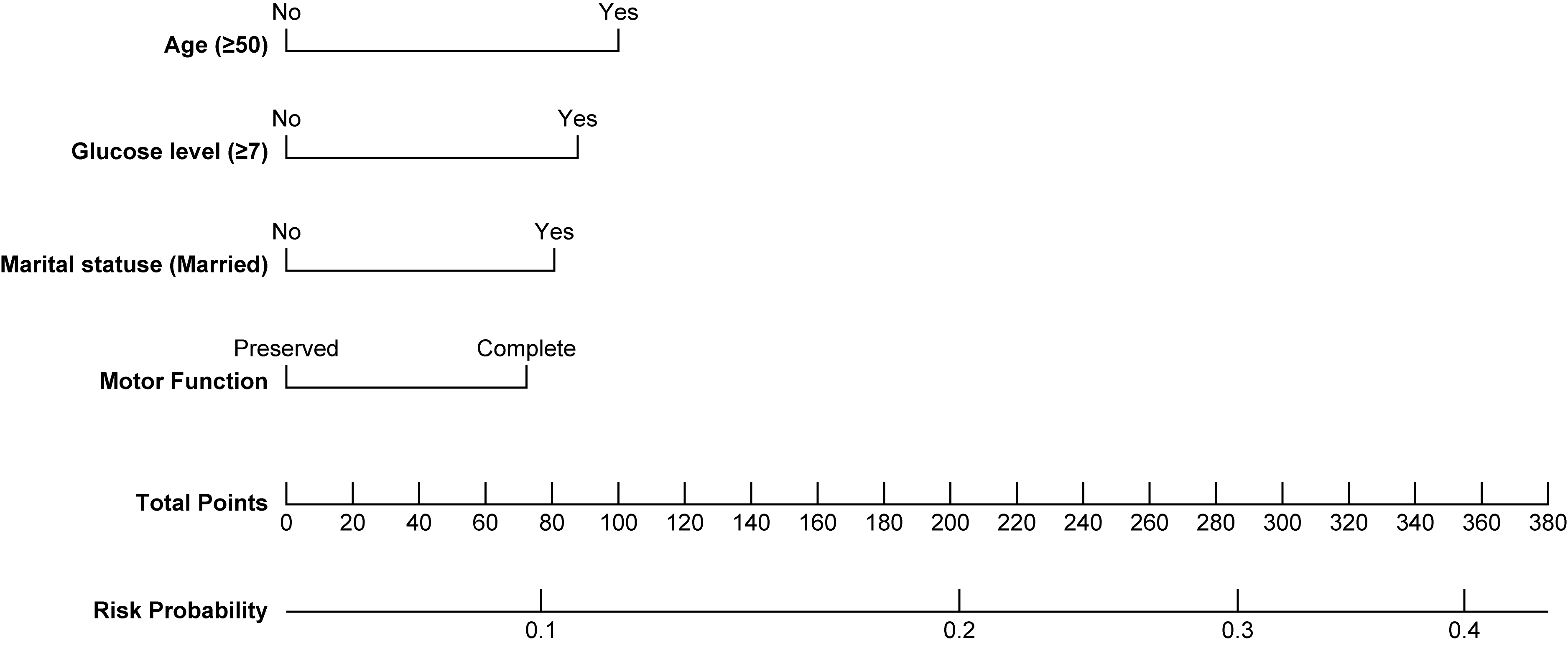

Supplement: S2 Fig — (TIF) [file pone.0344816.s004.tif]

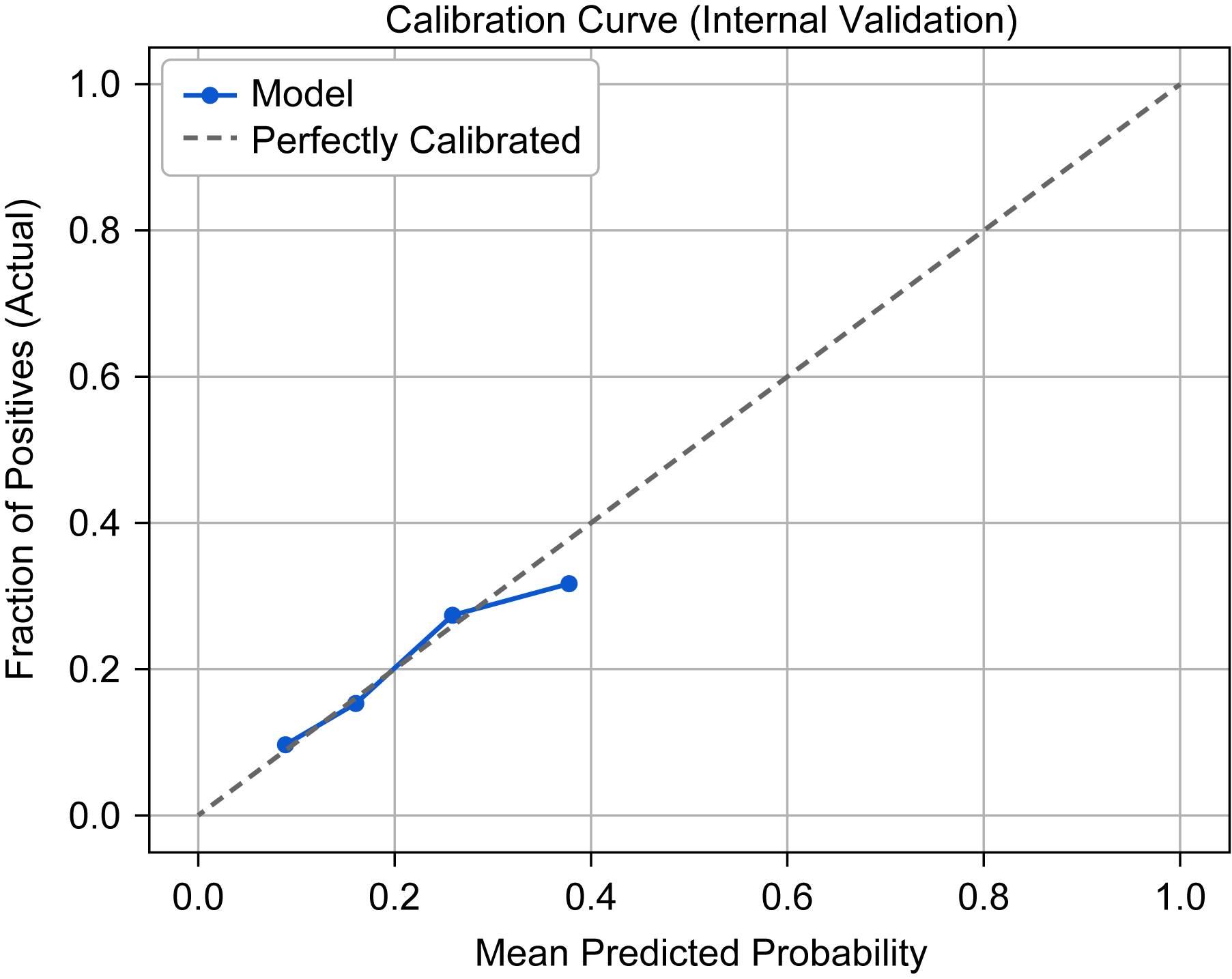

Supplement: S3 Fig — (TIF) [file pone.0344816.s005.tif]
